# Sulforaphane in Cancer Prevention and Therapy: A State-of-the-Art Review of Epidemiological Evidence, Molecular Mechanisms, and Translational Challenges

**DOI:** 10.3390/ijms27042028

**Published:** 2026-02-20

**Authors:** Jung Yoon Jang, Donghwan Kim, Na Kyeong Lee, Eunok Im, Nam Deuk Kim

**Affiliations:** 1 Department of Pharmacy, College of Pharmacy, Research Institute for Drug Development, Pusan National University, Busan 46241, Republic of Korea; jungyoon486@pusan.ac.kr (J.Y.J.); nklee@pusan.ac.kr (N.K.L.); 2 Functional Food Materials Research Group, Korea Food Research Institute, Wanju-gun 55365, Republic of Korea; kimd@kfri.re.kr; 3 Department of Pharmacy, Eson Geriatric Hospital, Ulsan 44955, Republic of Korea

**Keywords:** sulforaphane, chemoprevention, Nrf2–ARE pathway, epigenetic regulation, HDAC inhibition, cell death, clinical trials

## Abstract

Sulforaphane (SFN), an aliphatic isothiocyanate derived from cruciferous vegetables such as broccoli, has emerged as a chemopreventive dietary agent. SFN exerts multifaceted anticancer effects through the activation of the nuclear factor erythroid 2-related factor 2 (Nrf2)–antioxidant response element (ARE) pathways, inhibition of histone deacetylases (HDACs) and hypoxia-inducible factor-1α (HIF-1α), and regulation of apoptosis and autophagy. Epidemiological studies have consistently associated cruciferous vegetable intake with reduced cancer risk, while mechanistic research has elucidated the capacity of SFN to modulate redox balance, detoxification pathways, and epigenetic processes. Recent clinical trials have further demonstrated its potential to reduce carcinogenic biomarker levels and support metabolic detoxification. This review integrates evidence from epidemiological observations, molecular mechanisms, and clinical studies to provide a comprehensive understanding of the role of SFN in cancer prevention and therapy. Finally, translational challenges, including limited bioavailability, dose optimization, and standardization of broccoli-derived preparations, are discussed as critical factors for successfully translating SFN therapies from bench to bedside.

## 1. Introduction

Sulforaphane (SFN) is an aliphatic isothiocyanate derived from glucoraphanin, a major glucosinolate abundant in cruciferous vegetables such as broccoli, kale, and cauliflower. It is widely recognized as a bioactive dietary phytochemical with diverse biological activities [1]. SFN exerts pleiotropic cytoprotective, anti-inflammatory, and stress-adaptive effects by regulating redox homeostasis, mitochondrial function, immune responses, and cellular detoxification pathways, which collectively contribute to cellular stress control and homeostasis [2,3,4,5,6,7].

At a fundamental biological level, these diverse stress-adaptive effects converge on a limited number of central regulatory pathways. Among them, the nuclear factor erythroid 2-related factor 2 (Nrf2)–antioxidant response element (ARE) axis represents a principal molecular hub through which SFN orchestrates antioxidant and detoxification responses, together with the modulation of inflammatory and metabolic signaling networks, thereby enhancing cellular antioxidant capacity and detoxification systems [8]. Through engagement of these stress-response pathways, SFN has been implicated in the protection against cardiovascular, metabolic, and neurodegenerative disorders, underscoring its role as a systemic regulator of cellular stress responses and homeostasis [9,10,11,12,13]. Notably, comparable stress-adaptive and cytoprotective properties are shared to varying extents by other dietary isothiocyanates derived from cruciferous vegetables [14].

Representative compounds, including phenethyl isothiocyanate (PEITC), benzyl isothiocyanate (BITC), and allyl isothiocyanate (AITC), have demonstrated anticancer activities through overlapping mechanisms such as redox modulation, epigenetic regulation, and induction of programmed cell death, while SFN remains the most extensively investigated member owing to its favorable bioavailability, safety profile, and translational evidence [15,16].

At the mechanistic level, dysregulated redox balance, chronic inflammation, metabolic reprogramming, and impaired stress adaptation represent central hallmarks of carcinogenesis. Accordingly, these broad biological properties provide a strong mechanistic rationale for the relevance of SFN in cancer prevention and therapy [17]. Consistent with this concept, epidemiological studies have reported that sustained consumption of cruciferous vegetables is associated with a reduced risk of several malignancies, including lung, colorectal, prostate, and breast cancers, underscoring the translational potential of SFN in oncology [18,19,20,21]. These epidemiological findings have motivated subsequent experimental studies exploring the molecular and cellular mechanisms by which SFN may influence cancer initiation and progression [17]. These population-based observations provide a rationale for focusing on SFN as a representative bioactive compound mediating the chemopreventive effects of cruciferous vegetables.

In cancer-related contexts, SFN has been demonstrated to modulate multiple biological processes, including cellular detoxification pathways, epigenetic regulation, and programmed cell death, thereby influencing tumor growth and stress responsiveness [16,22,23,24,25,26,27,28,29]. Importantly, accumulating evidence indicates that the magnitude and nature of SFN-induced molecular responses are highly context-dependent and influenced by factors such as genetic background, gut microbiome composition, and dietary exposure patterns, highlighting substantial inter-individual variability in SFN bioactivity and translational outcomes [22].

Overall, growing experimental and translational evidence supports the view that SFN functions as a mechanism-based dietary compound with potential relevance to cancer prevention and supportive therapeutic strategies. Therefore, the present review aims to critically integrate current epidemiological observations, molecular mechanisms, preclinical findings, and emerging clinical data related to SFN, while highlighting key challenges and limitations that constrain its translational application in oncology. Unless otherwise specified, the findings discussed in this review are interpreted in an integrative manner across studies employing both purified SFN and food-derived formulations.

## 2. Literature Search Strategy and Study Selection

**Figure 1:**
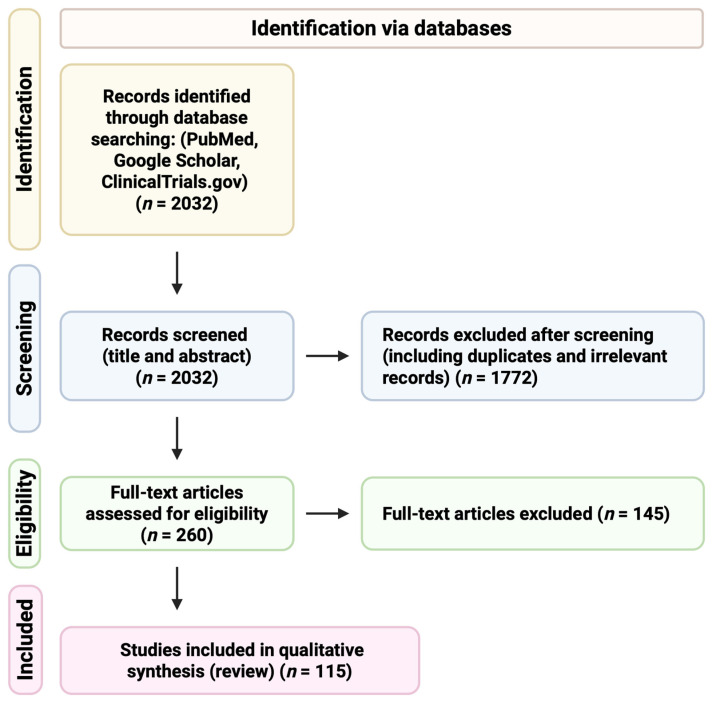
PRISMA-style flow diagram illustrating the literature search and study selection process. A focused literature search was conducted using PubMed, Google Scholar, and ClinicalTrials.gov to identify studies related to SFN in cancer prevention and therapy. Duplicate records were removed, and articles were screened based on relevance to epidemiological evidence, molecular mechanisms, preclinical models, and clinical outcomes. The study selection process followed the general PRISMA framework to ensure transparency and reproducibility of literature identification and screening [30]. The final qualitative synthesis included 115 studies. The figure was created using BioRender.com.

A focused literature search was conducted using PubMed, Google Scholar, and ClinicalTrials.gov to identify relevant studies examining SFN in cancer prevention and therapy. The search strategy combined the keyword “sulforaphane” with cancer-related and mechanistic terms. These included “cancer”, “tumor”, “Nrf2”, “epigenetic”, “cell death”, and “clinical trial”. For Google Scholar, the first 500 results sorted by relevance were screened due to the high volume of records retrieved. Duplicate records were identified and removed where applicable during the title and abstract screening process. Only peer-reviewed, English-language, full-text articles published up to December 2025 were considered eligible for inclusion. The overall study selection process is summarized in a PRISMA-style flow diagram (Figure 1).

## 3. Chemistry, Metabolism, and Bioavailability

SFN is a low-molecular-weight aliphatic isothiocyanate generated from the glucosinolate precursor glucoraphanin that is abundant in broccoli, Brussels sprouts, kale, and other cruciferous vegetables [31]. Glucoraphanin is relatively inert and water-soluble, whereas SFN is a highly reactive electrophile capable of covalently modifying protein thiols, a property that underlies its biological activity [32,33].

### 3.1. Glucoraphanin-to-SFN Conversion

In intact plant tissues, glucoraphanin and β-thioglucosidase myrosinase are spatially compartmentalized. However, tissue damage caused by cutting, chewing, or insect attack brings them into contact, leading to the rapid hydrolysis of glucoraphanin and the formation of SFN as the major isothiocyanate product [32,34]. The balance between SFN and alternative hydrolysis products such as SFN nitrile is influenced by factors such as pH, the presence of epithiospecifier proteins, and metal ions [35]. Cooking markedly affects this bioconversion, as plant myrosinase is heat-labile and is largely inactivated by prolonged boiling or microwaving, whereas brief steaming preserves substantial enzyme activity [36]. When plant myrosinase is inactivated, the conversion of glucoraphanin to SFN depends predominantly on the metabolic capacity of the gut microbiota, which possesses myrosinase-like activities capable of generating SFN and related metabolites in the colon [37].

### 3.2. Human Metabolism and the Mercapturic Acid Pathway

Once absorbed from the small intestine, SFN is predominantly metabolized via the mercapturic acid pathway, beginning with its rapid conjugation to glutathione (GSH), which is catalyzed by glutathione *S*-transferases to form the SFN–GSH conjugate [38]. SFN–GSH is subsequently processed by γ-glutamyltransferase, dipeptidases, and *N*-acetyltransferases to yield sequential cysteinyl-glycine (SFN–CysGly), cysteine (SFN–Cys), and *N*-acetylcysteine (SFN–NAC) conjugates, which represent the classical mercapturic acid pathway metabolites of SFN [39].

In human feeding studies utilizing broccoli sprout preparations, these SFN conjugates are readily detected in plasma and urine and typically account for the majority of the administered dose, with reports of 60–80% recovery of SFN equivalents in urine within 24 h after ingestion [37]. Importantly, as these mercapturic acid pathway metabolites represent the dominant in vivo forms of SFN, they serve as robust biomarkers for SFN exposure and pharmacokinetic behavior in clinical and nutritional intervention studies [38,40].

### 3.3. Determinants of SFN Bioavailability in Humans

**Figure 2:**
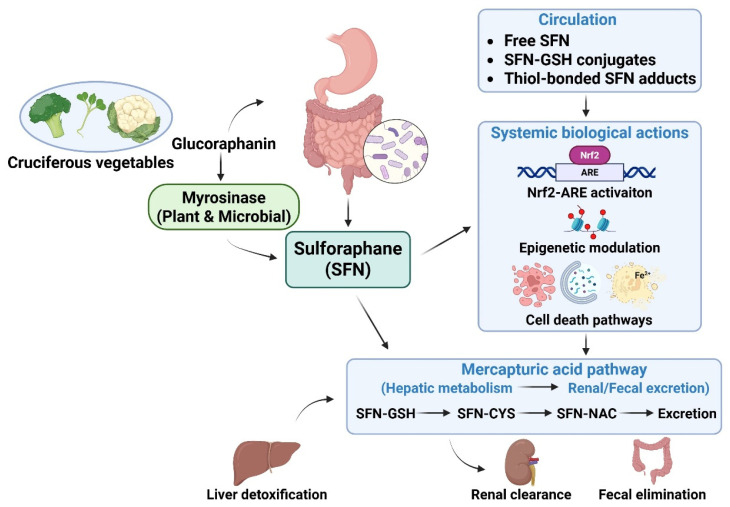
Metabolism and bioavailability of sulforaphane (SFN). SFN is generated from its precursor glucoraphanin that is abundant in cruciferous vegetables through the action of plant-derived or gut microbiota–derived myrosinase during food processing and digestion. Following intestinal absorption, SFN circulates in free and conjugated forms and is rapidly metabolized via the mercapturic acid pathway as a hepatic detoxification process through sequential conjugation with glutathione (SFN–GSH), cysteine (SFN–CYS), and *N*-acetylcysteine (SFN–NAC), leading to subsequent renal or fecal excretion. Inter-individual variability in SFN bioavailability is influenced by factors such as myrosinase activity, gut microbiome composition, and formulation stability, resulting in rapid systemic clearance and a relatively short biological half-life. Figure created using BioRender.com. CYS, cysteine; GSH, glutathione; NAC, *N*-acetylcysteine.

The human bioavailability of SFN is influenced by multiple interacting dietary and host factors. The presence of active myrosinase in the food matrix affects the conversion of glucoraphanin to SFN, while formulation type and gut microbiota composition can modulate absorption and metabolic processing [32,37,41]. Genetic polymorphisms in phase II enzymes such as glutathione *S*-transferases have also been reported to influence SFN conjugation and excretion, although their contribution appears relatively modest in human studies [42]. These factors are summarized schematically in Figure 2.

## 4. Molecular Mechanisms of SFN

**Figure 3:**
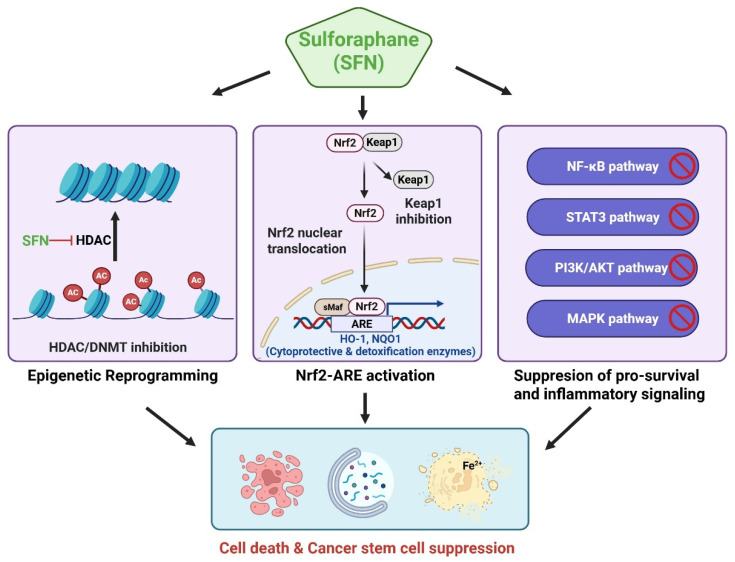
Multi-target anticancer mechanisms of sulforaphane (SFN). SFN exerts pleiotropic anticancer effects by concurrently modulating multiple interconnected molecular pathways. SFN activates the Nrf2–antioxidant response element (ARE) signaling axis by disrupting Keap1, resulting in the induction of cytoprotective and detoxification enzymes, including HO-1 and NQO1. In parallel, SFN suppresses major pro-survival and inflammatory signaling pathways such as nuclear factor kappa-light-chain-enhancer of activated B cells (NF-κB), signal transducer and activator of transcription 3 (STAT3), phosphoinositide 3-kinase (PI3K)/protein kinase B (AKT), and mitogen-activated protein kinase (MAPK). SFN also modulates epigenetic regulators, including histone deacetylases (HDACs) and DNA methyltransferases (DNMTs), thereby contributing to epigenetic reprogramming of aberrant transcriptional states in cancer cells. Collectively, these signaling alterations converge to induce multiple programmed cell death pathways, such as apoptosis, autophagy, and ferroptosis, and suppress cancer stem cell–associated properties, underpinning the broad anticancer potential of SFN across diverse tumor contexts. Figure created using BioRender.com. Ac, acetylation; AKT, protein kinase B; ARE, antioxidant response element; DNMTs, DNA methyltransferases; HDACs, histone deacetylases; HO-1, heme oxygenase-1; Keap1, Kelch-like ECH-associated protein 1; MAPK, mitogen-activated protein kinase; NF-κB, nuclear factor kappa-light-chain-enhancer of activated B cells; NQO1, NAD(P)H quinone dehydrogenase 1; Nrf2, nuclear factor erythroid 2–related factor 2; PI3K, phosphoinositide 3-kinase; SFN, sulforaphane; sMaf, small musculoaponeurotic fibrosarcoma protein; STAT3, signal transducer and activator of transcription 3.

SFN exerts anticancer effects through multiple molecular mechanisms, including the activation of the Nrf2–ARE antioxidant pathway, epigenetic regulation, and modulation of programmed cell death and oncogenic signaling. An integrated schematic summarizing these multilayered mechanisms is presented in Figure 3.

### 4.1. Nrf2–ARE Activation

Nrf2 is the master transcriptional regulator of cellular antioxidant defense, and SFN robustly activates this pathway by disrupting the negative regulator Kelch-like ECH-associated protein 1 (Keap1). SFN treatment in vitro and in vivo leads to the nuclear translocation of Nrf2 and upregulation of downstream cytoprotective genes, including those encoding detoxification enzymes and antioxidant proteins such as heme oxygenase 1 (HO-1), NAD(P)H quinone dehydrogenase 1 (NQO1), and glutathione-related enzymes [43]. Activation of the Nrf2–ARE pathway by SFN has been demonstrated to ameliorate oxidative and electrophilic stress in disparate tissues, thereby reducing cellular damage and suppressing processes that can initiate carcinogenesis or promote tumor progression [44]. In addition to its canonical antioxidant role, Nrf2 activation by SFN contributes to metabolic reprogramming that enhances cellular resilience under carcinogen-induced stress [16]. SFN-induced Nrf2 signaling upregulates enzymes involved in glutathione synthesis and nicotinamide adenine dinucleotide phosphate (NADPH) regeneration, thereby strengthening redox homeostasis and improving the detoxification capacity of premalignant and malignant cells [45]. Additionally, recent studies demonstrate that SFN-mediated Nrf2 activation modulates inflammatory signaling by suppressing nuclear factor kappa-light-chain-enhancer of activated B cells (NF-κB)-driven transcription, thereby reducing the production of pro-inflammatory cytokines implicated in tumor initiation and progression [46]. Although Nrf2 activation can exhibit context-dependent effects in established tumors, preclinical evidence consistently indicates that SFN-driven Nrf2 signaling supports chemopreventive activity by maintaining genomic stability and mitigating oxidative damage during early carcinogenesis [47]. Importantly, accumulating evidence indicates that the biological consequences of Nrf2 activation are highly dependent on its magnitude, duration, and cellular context. Transient, diet-derived activation by SFN predominantly confers cytoprotective and chemopreventive effects, in contrast to the sustained oncogene-driven Nrf2 hyperactivation observed in certain advanced malignancies [16,47]. In established tumors, constitutive Nrf2 signaling is most frequently driven by loss-of-function mutations in *KEAP1* that disrupt normal Nrf2 degradation and result in persistent pathway activation. Such *KEAP1*-deficient cancers exhibit enhanced antioxidant capacity, metabolic reprogramming, and increased tolerance to oxidative and therapeutic stress, thereby promoting tumor cell survival and resistance to chemotherapy and radiotherapy. Mechanistically, chronic Nrf2 activation in this context upregulates detoxification enzymes, redox-balancing systems, and stress-adaptive metabolic pathways, conferring selective growth advantages under therapeutic pressure [48]. Accordingly, in tumors with pre-existing *KEAP1* dysfunction and constitutive Nrf2 activation, additional pharmacological or dietary stimulation of the Nrf2 pathway, including SFN supplementation, may offer limited therapeutic benefit and may theoretically reinforce stress-adaptive survival mechanisms in cancer cells [47,48]. In this context, these considerations underscore the importance of context- and genotype-aware application of SFN in oncology, thereby supporting its preferential positioning in cancer prevention, early interception, or biomarker-guided adjunctive strategies rather than indiscriminate use in advanced, *KEAP1*-altered malignancies [17,22,49].

### 4.2. Epigenetic Modulation

SFN is a well-characterized dietary HDAC inhibitor, and multiple studies have demonstrated that SFN decreases HDAC activity and increases histone H3/H4 acetylation in cancer cells [50]. Notably, SFN preferentially targets class I histone deacetylases, particularly HDAC1, HDAC2, and HDAC3, which are central mediators of transcriptional repression of tumor suppressor genes in cancer cells [24,50,51,52]. Mechanistically, SFN and its thiol-reactive metabolites are proposed to impair the Zn^2+^-dependent catalytic activity of histone deacetylases (HDACs) through modification of critical cysteine residues within their catalytic domains, providing a mechanistic explanation for the increased acetylation of histone H3 and H4 [24,50,53]. SFN-mediated HDAC inhibition leads to chromatin relaxation and reactivation of tumor-suppressive genes such as p21 and *BAX*, promoting cell cycle arrest and apoptosis in colorectal and prostate cancer models [51].

Beyond histone acetylation, accumulating evidence indicates that SFN also modulates DNA methylation patterns, further contributing to epigenetic reprogramming in cancer cells. In prostate cancer transgenic adenocarcinoma of mouse prostate (TRAMP) C1 cells, SFN demethylates CpG sites within the *NRF2* promoter and reduces DNA methyltransferase (DNMT)1 binding, resulting in the restoration of Nrf2 transcriptional activity [54]. SFN-induced inhibition of DNMT has also been reported in colon epithelial cells, where promoter demethylation of cytoprotective genes enhances antioxidant and detoxification capacities [55]. Collectively, these findings indicate that SFN targets multiple epigenetic layers, including HDACs, DNMTs, and chromatin architecture, to reverse cancer-associated epigenetic abnormalities and shift transcriptional programs toward a tumor-suppressive phenotype [53].

### 4.3. Cell Death and Anticancer Signaling

Epigenetic reprogramming induced by SFN establishes a permissive transcriptional landscape that facilitates the activation of genes involved in programmed cell death and metabolic stress responses. Accordingly, SFN-induced apoptosis, autophagy, and ferroptosis represent coordinated downstream consequences of specific molecular target modulation rather than nonspecific cytotoxic effects [16,24,49,53].

SFN has been demonstrated to induce intrinsic apoptosis in multiple tumor types. At the molecular level, SFN promotes mitochondrial apoptosis through stabilization of p53, upregulation of the pro-apoptotic protein Bax, mitochondrial cytochrome *c* release, and subsequent activation of caspase-9 and caspase-3 [25,56,57]. In oral squamous cell carcinoma, SFN induces mitochondrial cytochrome *c* release, activates caspase-9 and caspase-3, and increases p53 expression, culminating in robust and dose-dependent apoptotic cell death [56]. Consistent with these observations, SFN promoted apoptotic cell death in pancreatic cancer models, where it suppressed proliferation and metastasis and induced G2/M cell cycle arrest in PANC-1 pancreatic carcinoma cells, further supporting its role as an apoptosis-modulating agent in gastrointestinal malignancies [58].

Beyond apoptosis, SFN modulates autophagy in a context-dependent manner. SFN regulates autophagy primarily through metabolic stress–responsive signaling pathways involving activation of AMP-activated protein kinase (AMPK) and inhibition of mammalian target of rapamycin (mTOR), thereby modulating downstream autophagy-initiating complexes such as ULK1 [16,26,59]. In esophageal squamous cell carcinoma cells, SFN induces protective autophagy via reactive oxygen species (ROS)-dependent AMPK/mTOR signaling, and pharmacological inhibition of autophagy enhances SFN-induced apoptosis, suggesting that autophagy may partially attenuate SFN cytotoxicity under these conditions [59]. In contrast, in non-small cell lung cancer, SFN suppresses autophagy by downregulating fatty acid synthase and perturbing tumor lipid metabolism, thereby enhancing susceptibility to apoptosis and limiting malignant progression [60]. Taken together, these findings indicate that the effect of SFN on autophagy reflects a context-dependent balance between cytoprotective and pro-death signaling pathways in various tumor types.

More recently, accumulating evidence indicates that SFN participates in non-apoptotic cell death pathways, particularly in ferroptosis. At the molecular level, SFN-induced ferroptosis is characterized by suppression of the cystine/glutamate antiporter subunit SLC7A11, depletion of intracellular glutathione, inhibition of glutathione peroxidase 4 (GPX4), and subsequent iron-dependent lipid peroxidation [27,61,62]. In acute myeloid leukemia cells, SFN induces lipid peroxidation, iron accumulation, and GPX4 suppression in a dose-dependent manner, resulting in the concurrent activation of apoptotic and ferroptotic cell death programs [27]. SFN also induces ferroptosis in small cell lung cancer and promotes sirtuin-3–dependent ferroptosis in colorectal cancer through AMPK-mediated metabolic signaling [61,62].

### 4.4. Additional Antitumor Mechanisms of SFN

Beyond its established roles in Nrf2–ARE pathway activation, epigenetic regulation, and cell death induction, SFN exerts anticancer effects through additional mechanisms that contribute to tumor growth inhibition and therapeutic sensitivity [16,24,53]. SFN has been reported to disrupt cancer cell metabolic reprogramming, particularly by suppressing lipid metabolism through downregulation of fatty acid synthase (FASN), thereby impairing mitochondrial function and bioenergetic homeostasis in tumor cells [60]. SFN also modulates the tumor microenvironment by attenuating tumor-promoting inflammation and reducing pro-inflammatory cytokine production, which limits inflammation-driven cancer progression [63]. Additionally, SFN suppresses epithelial–mesenchymal transition (EMT), resulting in reduced invasiveness and metastatic potential of cancer cells. Consistent with these effects, SFN has also been reported to promote cellular differentiation programs, contributing to the restoration of more differentiated epithelial phenotypes and reinforcing its chemopreventive activity [64]. Furthermore, SFN exhibits anti-angiogenic activity by inhibiting hypoxia-related signaling pathways, including downregulation of hypoxia-inducible factor-1α (HIF-1α) and vascular endothelial growth factor (VEGF), thereby restricting tumor neovascularization [29]. Moreover, SFN inhibits multiple oncogenic and prosurvival signaling pathways, including NF-κB, signal transducer and activator of transcription 3 (STAT3), phosphoinositide 3-kinase (PI3K)/protein kinase B (AKT), and mitogen-activated protein kinase (MAPK), thereby suppressing proliferation, inflammation, invasion, and metastasis [57,65]. Specifically, SFN downregulates oncogenic regulatory circuits such as the STAT3–CKMT2-AS1 axis and attenuates inflammatory cytokine signaling, including interleukin-1 beta (IL-1β)–induced interleukin-6 (IL-6) expression via ROS-dependent MAPK/activator protein-1 (AP-1) pathways, reinforcing its broad antitumor activity beyond direct induction of cell death [63,66,67].

## 5. Preclinical and Clinical Evidence

A growing body of preclinical and clinical evidence supports the anticancer potential of SFN across diverse experimental models. Consistent with its molecular mechanisms, SFN has been demonstrated to inhibit cell proliferation, reduce tumor growth, impair metastatic potential, and suppress malignant phenotypes in multiple cancer systems [68,69].

### 5.1. Preclinical Summary

In the SFN oncology literature, prostate, breast, colorectal, and lung cancers have been among the most frequently investigated indications in preclinical studies. Therefore, Table 1 was organized to reflect the relative breadth of the available evidence and improve readability.

#### 5.1.1. Prostate Cancer

In androgen-responsive prostate cancer models, including LNCaP (lymph node carcinoma of the prostate) and 22Rv1 cells, SFN suppresses key lipogenic regulators such as acetyl-CoA carboxylase 1 (ACC1), FASN, carnitine palmitoyltransferase 1A (CPT1A), and sterol regulatory element-binding protein 1 (SREBP1), supporting a mechanism centered on the inhibition of fatty acid synthesis and metabolic reprogramming. Consistent with these in vitro findings, SFN administration in the TRAMP model led to a marked reduction in lipid-related metabolites (such as total free fatty acids and phospholipids) and energy-associated intermediates (e.g., acetyl-CoA and ATP), concomitantly activating Nrf2–associated cytoprotective signaling, indicating a coordinated metabolic and stress response axis in vivo [70]. Beyond metabolic regulation, SFN also targets prostate cancer stem-like properties by decreasing tumorsphere formation and downregulating stemness-associated markers, including cluster of differentiation 44 (CD44) and aldehyde dehydrogenase 1 (ALDH1), accompanied by suppression of Wnt/β-catenin and hedgehog signaling and induction of pro-apoptotic effects [71].

#### 5.1.2. Breast Cancer

In triple-negative breast cancer cells (MDA-MB-231), SFN attenuates metastatic potential by targeting the rapidly accelerated fibrosarcoma (RAF)/mitogen-activated protein kinase kinase (MEK)/extracellular signal-regulated kinase (ERK) signaling cascade, as evidenced by the reduced expression of RAF family proteins and decreased activation of downstream MEK and ERK [68]. In addition to suppressing invasion-associated signaling, SFN inhibits breast cancer cell proliferation in both MDA-MB-231 and ZR-75-1 estrogen receptor-positive breast cancer cells by inducing mitotic delay, accompanied by modulation of key cell-cycle regulators, including cyclin B1, cell division cycle 2 (CDC2), and cell division cycle 25C (CDC25C), and activation of cyclin-dependent kinase 5 regulatory subunit 1 (CDK5R1)/cyclin-dependent kinase 5 (CDK5)-associated signaling, ultimately resulting in G2/M arrest and apoptosis [72]. Beyond proliferative and migratory control, SFN also targets breast cancer stem-like phenotypes by reducing mammosphere formation and cancer stem cell (CSC)-enriched populations in vitro and by suppressing tumor growth in xenograft models, concomitant with the downregulation of Cripto-1 (teratocarcinoma-derived growth factor 1, TDGF1)-related stemness markers, suggesting that SFN may limit tumor growth as well as recurrence- and resistance-associated subpopulations in breast cancer [73].

#### 5.1.3. Colorectal Cancer

In HCT116 colorectal cancer cells, SFN promotes ferroptosis-associated features, including suppression of solute carrier family 7 member 11 (*SLC7A11*), increased ROS production, enhanced lipid peroxidation, and altered intracellular iron levels, that are mechanistically linked to the activation of sirtuin 3 (SIRT3)–AMPK–mTOR signaling axis [62]. Beyond the direct regulation of ferroptotic cell death, SFN also modulates inflammatory signaling in HT-29 cells by suppressing IL-1β–induced IL-6 transcription and secretion through the attenuation of ROS accumulation and inhibition of p38 MAPK–AP-1 and STAT3 activation, accompanied by reduced invasion and migration phenotypes [63]. In addition to cell death induction and inflammatory control, SFN inhibits proliferation and motility in HT-29 and SW480 colorectal cancer models while engaging an extracellular signal-regulated kinase (ERK)–Nrf2–UDP-glucuronosyltransferase family 1 member A (UGT1A)–dependent metabolic detoxification axis together with cell-cycle arrest and apoptosis-associated signatures, supporting a multi-layered mechanism that integrates signaling suppression with enhanced detoxification capacity [74].

### 5.2. Clinical Studies

As of December, 2025, a search of the ClinicalTrials.gov registry using the keyword “sulforaphane” identified approximately 80–90 registered clinical studies, of which approximately 20 trials were related to cancer prevention or therapeutic intervention. These cancer-focused studies primarily evaluated SFN- or SFN-rich preparations in prostate, breast, lung, and colorectal cancer settings, as well as in cancer risk- or biomarker-based intervention trials. Among these registered studies, clinical trials with available outcome data or reported results are selectively summarized in Table 2 to highlight the current state of clinical evidence supporting the anticancer potential of SFN.

Clinical evidence for the anti-cancer effects of SFN has primarily been derived from prostate cancer–related clinical studies. Early phase clinical trials employing SFN or SFN-rich broccoli sprout preparations have demonstrated favorable modulation of prostate cancer–associated biomarkers, including a reduction in prostate-specific antigen (PSA) levels in a subset of patients and increased urinary excretion of SFN metabolites, supporting systemic bioavailability and target engagement [78,79,80,81]. Additionally, pre-surgical or biopsy window studies have further confirmed that oral administration of SFN-rich preparations leads to a measurable accumulation of SFN metabolites in prostate tissue, indicating effective tissue penetration in humans [80,81].

In breast cancer-related clinical studies, SFN has been primarily evaluated using presurgical window-of-opportunity designs. These trials demonstrated that short-term administration of SFN-rich broccoli sprout preparations resulted in decreased HDAC activity and modulation of cancer-associated biomarkers in breast tissue, supporting the epigenetic activity of SFN in a clinical context [82,83,84,85]. Although these studies were not designed to evaluate long-term clinical outcomes, they provide supportive mechanistic evidence that SFN exposure is associated with the modulation of cancer-related molecular endpoints in human breast tumors.

Clinical investigations of lung cancer risk have largely focused on cancer risk reduction and biomarker modulation, rather than direct therapeutic efficacy. In individuals at an elevated risk of lung cancer, including former smokers, SFN-rich interventions were evaluated primarily through histopathological and proliferation-related biomarkers, including changes in the bronchial dysplasia index and modulation of the cell proliferation marker Ki-67 in the bronchial epithelium, with modest biomarker modulation but without definitive between-group efficacy signals [86]. These findings support the potential role of SFN in modulating biological processes associated with early carcinogenesis rather than promoting the regression of established tumors.

Taken together, the available clinical studies suggest that SFN is well tolerated in humans and can modulate cancer-related biomarkers in multiple organ sites. However, current clinical evidence is largely derived from small-scale, short-term studies relying on surrogate endpoints, and this limits definitive conclusions regarding the preventive or therapeutic efficacy of SFN in oncology.

## 6. Challenges, Translational Barriers, and Future Perspectives

The bioavailability of active SFN in humans is highly variable and influenced by multiple dietary and host-related factors, including the food matrix, myrosinase activity, individual gut microbiome composition, and formulation stability [32,87,88,89]. Additionally, optimal dosing regimens, long-term safety data, and standardized clinical endpoints for cancer prevention and therapy remain incompletely characterized, complicating consistent clinical translation [22,49]. Collectively, these limitations continue to hinder the consistent translation of SFN from bench to bedside [57,90,91].

### 6.1. Bioavailability-Related Variability and Translational Barriers

One of the primary translational barriers to the clinical application of SFN is substantial inter-individual variability in systemic exposure, even when similar doses are administered [41,90,92]. Notably, the dietary and host-related factors described above, including differences in glucoraphanin content, myrosinase activity, food processing conditions, and gut microbiota composition, together represent a major translational barrier [32,92,93]. As inconsistent SFN generation and stability directly compromise reproducibility across clinical studies, recent reviews emphasize that controlling enzymatic conversion and improving SFN stabilization are critical for achieving reliable and comparable bioavailability profiles [91,94,95]. Accordingly, the development of optimized extraction, stabilization, and manufacturing strategies is a prerequisite for advancing SFN from dietary supplementation to clinically deployable interventions [93,96].

### 6.2. Biomarker-Guided and Mechanism-Driven Clinical Trial Design

Given the predominance of biomarker-based outcomes in existing SFN trials, future clinical studies should integrate pharmacokinetic and target-engagement biomarkers, such as urinary dithiocarbamate metabolites or tissue-level molecular readouts, to support dose optimization and mechanistic interpretation [91,97]. This biomarker-centered approach is particularly relevant in cancer prevention and early interception settings, where long-term clinical endpoints such as cancer incidence or survival are impractical to assess within a feasible study period [17,22,28]. Under these circumstances, surrogate molecular changes function as the principal indicators of biological activity, underscoring the importance of carefully selecting and validating biomarkers [91]. Accordingly, the harmonization of biomarker panels and outcome definitions across studies is essential to enable cross-trial comparisons and improve the interpretability and translational value of emerging clinical evidence [17,91].

### 6.3. Advanced Delivery Systems and Combination Strategies

A major limitation of the therapeutic application of SFN is its physicochemical instability and rapid metabolism, which limit sustained systemic and tissue-level exposure following conventional administration [98]. Free SFN is chemically labile and undergoes rapid conjugation via the mercapturic acid pathway, resulting in a short biological half-life, pronounced inter-individual pharmacokinetic variability, and limited reproducibility of systemic exposure in clinical settings [37,38,41]. To address these limitations, a range of advanced delivery technologies has been developed with the primary objective of improving SFN stability, bioaccessibility, and pharmacokinetic consistency rather than simply increasing intrinsic anticancer potency [94].

Advanced delivery approaches, including microencapsulation, enzyme-stabilized formulations, and nanotechnology-based carriers, have been explored to improve SFN stability, bioaccessibility, and pharmacokinetic consistency, with preclinical studies indicating enhanced intracellular uptake and tissue accumulation compared to free SFN [94,96,99]. Microencapsulation strategies, typically employing protein- or polysaccharide-based matrices, protect SFN from premature degradation during food processing and gastrointestinal transit, enabling delayed release and improved bioaccessibility [96]. Enzyme-stabilized formulations, most commonly based on glucoraphanin combined with active myrosinase, provide more controlled and reproducible in situ SFN generation, leading to improved systemic exposure and reduced inter-individual variability relative to free SFN preparations [32,88].

Nanotechnology-based delivery systems, including liposomes and polymeric nanoparticles, have demonstrated the capacity to prolong circulation time, enhance cellular uptake, and increase tissue-level accumulation of SFN in preclinical models; however, their clinical translation remains limited by scalability, regulatory complexity, and long-term safety considerations [99,100]. From a pharmacokinetic perspective, these advanced delivery systems consistently outperform free SFN with respect to formulation stability, exposure duration, and reproducibility of absorption profiles, as summarized in Table 3 [94].

Nevertheless, delivery optimization alone is unlikely to establish SFN as a primary anticancer therapy, given the ongoing challenges related to scalability, long-term safety, regulatory complexity, and interstudy heterogeneity [100]. Accordingly, the therapeutic potential of SFN is realized using combination strategies rather than monotherapies [101]. By modulating redox homeostasis, stress-response signaling, epigenetic regulation, and cancer stem cell–associated pathways implicated in therapeutic resistance, SFN provides a strong mechanistic rationale for its use as an adjunctive agent rather than a standalone cytotoxic compound [49,102]. These pleiotropic mechanisms are particularly relevant to overcoming therapy-associated resistance and enhancing treatment responsiveness, thereby supporting the integration of SFN into combination regimens with cytotoxic chemotherapy, targeted therapies, or radiotherapy [17,101,102].

### 6.4. Long-Term Safety Considerations for Chronic SFN Administration

Although SFN has demonstrated favorable short-term tolerability in clinical studies, its application in cancer prevention and chronic intervention settings requires careful evaluation of long-term safety. Existing human trials, which have largely been of limited duration, report predominantly mild and transient adverse effects such as gastrointestinal discomfort or headache, without evidence of serious systemic toxicity [22,91,103]. Preclinical toxicological data suggest a broad safety margin at doses relevant to dietary or supplemental exposure; however, SFN responses appear hormetic and dose-dependent, while exerting biphasic biological effects through which low or intermittent exposure activates adaptive cytoprotective and detoxification pathways; however, sustained or high-dose exposure may lead to context-dependent adverse or off-target responses through prolonged modulation of redox- and stress-response signaling [90,98,104]. Given that SFN undergoes extensive hepatic metabolism and renal excretion, altered pharmacokinetics may occur in individuals with impaired liver or kidney function, warranting cautious dose selection and clinical monitoring in these populations [37,90]. While current evidence supports the general safety of short-term SFN administration, recognition of its hormetic behavior underscores the need for careful dose optimization and exposure duration control; thus, well-designed studies incorporating longer follow-up and standardized safety endpoints are required to establish its long-term safety profile and support its translational use in preventive or chronic clinical settings [17,22,91].

## 7. Conclusions

SFN is a well-characterized dietary isothiocyanate with substantial experimental and clinical evidence supporting its role in cancer prevention and biological modulation of tumor-related processes. Preclinical studies have consistently demonstrated that SFN exerts anticancer effects by regulating redox homeostasis, epigenetic mechanisms, and stress-response signaling, thereby influencing key processes involved in carcinogenesis.

Human studies further indicate that SFN, predominantly administered as SFN-rich preparations, is generally well tolerated and is associated with the modulation of cancer-relevant molecular biomarkers in clinical settings, despite its low and variable systemic bioavailability. Although current clinical evidence is largely derived from early-phase and biomarker-driven trials rather than from definitive outcome studies, these findings provide important proof-of-mechanism for the biological activity of SFN in humans.

Collectively, these data suggest that SFN is unlikely to function as a standalone anticancer drug. However, its translational potential is considered more appropriate within cancer prevention frameworks or as a supportive mechanism-based intervention that complements established therapeutic strategies. Future progress in this field will depend on the development of standardized formulations with reproducible bioavailability, biomarker-guided clinical trial designs, and rigorous integration of mechanistic endpoints.

Thus, SFN is a scientifically substantiated dietary phytochemical with significant potential for cancer prevention and translational oncology research. Continued efforts to align formulation science, mechanistic understanding, and clinical study design are essential to define its optimal role in evidence-based cancer intervention paradigms.

## Figures and Tables

**Table 1:** Summary of preclinical evidence for the anticancer effects of SFN across multiple cancer types.

Cancer Type	Models (In Vitro/In Vivo)	Key Findings	Major Mechanisms	Refs.
Prostate cancer	LNCaP, 22Rv1 cells (in vitro)	↓: ACC1, FASN, CPT1A, SREBP1	↓: fatty-acid synthesis pathway	[70]
	TRAMP mice (in vivo)	↓: total free fatty acids, total phospholipids, acetyl-CoA, ATP, neutral lipid droplets	↓: fatty-acid metabolism ↑: Nrf2-mediated cytoprotective signaling	[70]
	PC-3 CSC-like cells (in vitro)	↓: tumor sphere formation, PCSC markers (CD44, ALDH1, Oct4, Nanog)	↓: Wnt/β-catenin & Hedgehog pathways ↑: apoptosis	[71]
Breast cancer	MDA-MB-231 (in vitro)	↓: RAF family proteins (ARAF, BRAF, and CRAF), MEK, p-ERK	↓: metastasis signaling	[68]
	MDA-MB-231, ZR-75-1 cells (in vitro)	↓: Cyclin B1, CDC2, CDC25C ↑: MPM-2 activity, CDK5R1, CDK5	↓: proliferation ↑: apoptosis, mitotic arrest (G2/M)	[72]
	MDA-MB-231-Luc-D3H1 CSC-like cells (in vitro)	↓: mammosphere formation (1, 2, 3), CSC population (CD44^+^/CD24^−^/CD49f^+^), CSC-associated stemness signaling (CR1, CR3, WNT3, NOTCH4, FOXD3)	↓: stemness pathway, self-renewal capacity	[73]
	MDA-MB-231-Luc-D3H1 xenograft (in vivo)	↓: tumor volume, Cripto-1 protein expression (CR1/CR3; IHC trend)	↓: CSC-driven tumor growth	[73]
Colorectal cancer	HCT116 cells (in vitro)	↓: cell viability, SLC7A11 ↑: ROS, MDA, intracellular iron	↑: SIRT3–AMPK–mTOR axis–mediated ferroptosis	[62]
	HT-29 cells (in vitro)	↓: IL-6 mRNA, IL-6 secretion, IL-6 promoter activity, ROS, p-p38 MAPK, p-STAT3, p-c-Jun/AP-1, invasion, migration	↓: ROS–p38 MAPK–AP-1/STAT3 inflammatory signaling	[63]
	HT-29, SW480 cells (in vitro)	↓: cell viability, colony formation, EdU proliferation, migration (wound healing & transwell) ↑: ROS, G0/G1 arrest, apoptosis, p-ERK/ERK ratio, Nrf2, UGT1A	↑: ERK–Nrf2–UGT1A metabolic detoxification pathway	[74]
Lung cancer	A549, H460 CD133^+^ cells (in vitro)	↓: cell viability, tumorsphere formation, SHH signaling (Shh, Smo, Gli1), PHC3	↓: Sonic Hedgehog signaling and PHC3-associated self-renewal	[75]
Pancreatic cancer	Mia PaCa-2 cells (in vitro)	↓: cell viability, p-NF-κB p65, NF-κB p65, NF-κB p50, c-Myc, BCL-2 ↑: p-GSK-3β, β-catenin, cleaved caspase-3 and PARP	↑: apoptosis via modulation of the GSK-3β/β-catenin pathway	[76]
Bladder cancer	T24, UMUC3 cells (in vitro)	↓: pseudopodia formation (lamellipodia, filopodia, invadopodia), cell migration and invasion, CTTN (cortactin), WASL, ACTR2/ARP2, ATP production, ECAR, OCR	↓: actin nucleation–driven pseudopodia formation (CTTN–WASL–ARP2/3, AKT1-ATP axis)	[77]
	T24-luc tail-vein metastasis model (in vivo)	↓: lung metastatic burden, metastatic tumor growth, Actr2s/Arp2 and CTTN expression in metastatic lung tissue	↓: pseudopodia-dependent metastatic colonization	[77]

ACC1, acetyl-CoA carboxylase 1; ACTR2 (ARP2), actin-related protein 2; AKT1, AKT serine/threonine kinase 1; ALDH1, aldehyde dehydrogenase 1; AP-1, activator protein 1; ARP2/3, actin-related protein 2/3 complex; ATP, adenosine triphosphate; BCL-2, B-cell lymphoma 2; CD133, prominin-1; CD24, cluster of differentiation 24; CD44, cluster of differentiation 44; CD49f, integrin alpha-6 (*ITGA6*); CDC2 (CDK1), cyclin-dependent kinase 1; CDC25C, cell division cycle 25C; CDK5, cyclin-dependent kinase 5; CDK5R1, cyclin-dependent kinase 5 regulatory subunit 1; c-Myc, *MYC* proto-oncogene, bHLH transcription factor; CPT1A, carnitine palmitoyltransferase 1A; CR1, Cripto-1 (TDGF1); CR3, Cripto-3 (TDGF3); CRAF, RAF1 proto-oncogene serine/threonine kinase; CSC, cancer stem cell; CTTN, cortactin; ECAR, extracellular acidification rate; FASN, fatty acid synthase; FOXD3, forkhead box D3; Gli1, glioma-associated oncogene homolog 1; GSK-3β, glycogen synthase kinase-3 beta; G0/G1, cell-cycle phase G0/G1; IHC, immunohistochemistry; MDA, malondialdehyde; MEK, mitogen-activated protein kinase kinase; MPM-2, mitotic protein monoclonal 2 epitope; Nanog, Nanog homeobox; NF-κB, nuclear factor kappa-light-chain-enhancer of activated B cells; NOTCH4, notch homolog 4; Nrf2, nuclear factor erythroid 2–related factor 2; OCR, oxygen consumption rate; Oct4, octamer-binding transcription factor 4; PARP, poly(ADP-ribose) polymerase; PCSCs, prostate cancer stem-like cells; p-ERK, phosphorylated extracellular signal-regulated kinase; PHC3, polyhomeotic homolog 3; p-p38 MAPK, phosphorylated p38 mitogen-activated protein kinase; p-STAT3, phosphorylated signal transducer and activator of transcription 3; Shh, sonic hedgehog; *SLC7A11*, solute carrier family 7 member 11; Smo, smoothened; SREBP1, sterol regulatory element-binding protein 1; transwell, transwell migration assay; TRAMP, transgenic adenocarcinoma of mouse; UGT1A, UDP-glucuronosyltransferase family 1 member A; WNT3, Wnt family member 3; prostate; 1, primary; 2, secondary; 3, tertiary; ↑, increase; ↓, decrease.

**Table 2:** Clinical trials evaluating SFN in cancer and cancer-risk settings with reported outcomes.

Cancer Type	Phase	Study Design	Key Baseline Characteristics	Main Outcomes	Clinical Trial ID	Refs.
Prostate cancer	Phase 2	Single-arm interventional study	*n* = 20; biochemical recurrence; SFN-rich broccoli sprout extract	≥50% PSA decline achieved in 1 of 20 patients	NCT01228084	[78,79]
	Not applicable	Interventional biopsy window study	*n* = 98; men scheduled for prostate biopsy; SFN-rich broccoli sprout extract capsules	Increase in urinary SFN metabolites	NCT01265953	[80,81]
Breast cancer	Phase 2	Randomized, placebo-controlled presurgical window study	*n* = 54; presurgical breast cancer; SFN-yielding broccoli sprout preparation	HDAC activity decreased in breast tissue	NCT00843167	[82,83,84]
	Phase 2	Interventional breast tissue biomarker study	*n* = 34; women scheduled for breast biopsy; SFN-rich broccoli sprout preparation	Cancer-related biomarkers showed changes in breast tissue	NCT00982319	[85]
Lung cancer risk (former smokers)	Phase 2	Randomized, placebo-controlled lung tissue biomarker study	*n* = 43; former smokers at high lung cancer risk; SFN	Modulation of bronchial dysplasia index and Ki-67 proliferation marker over 12 months	NCT03232138	[86]

HDAC, histone deacetylase; PSA, prostate-specific antigen; SFN, sulforaphane.

**Table 3:** Pharmacokinetic comparison of free SFN and advanced delivery systems.

System	Representative Example	Stability	PK Features	Key Advantage	Key Limitation	Refs.
Free SFN	Pure SFN	Low	Short half-life; high inter-individual variability	Simple formulation	Instability; rapid metabolism	[37,38,41]
Microencapsulation	Protein-based matrix	↑	Delayed release; increased bioaccessibility	Improved stability	Limited human PK data	[96]
Enzyme-stabilized formulations	Glucoraphanin + active myrosinase	↑↑	Reproducible in situ SFN generation	Reduced PK variability	Enzyme dependence	[32,88]
Nanotechnology-based carriers	Liposomes/polymeric nanoparticles	↑↑↑	Prolonged exposure; enhanced uptake *	Targeted delivery potential	Limited clinical translation	[99,100]

PK, pharmacokinetic; SFN, sulforaphane: *, evidence derived primarily from preclinical studies; ↑, relative increase compared with free SFN; ↑↑, moderate increase; ↑↑↑, marked increase.

## Data Availability

No new data were created or analyzed in this study. Data sharing is not applicable to this article.

## Abbreviations

The following abbreviations are used in this manuscript:

AKT	Protein kinase B
AMPK	AMP-activated protein kinase
AP-1	Activator protein 1
ARE	Antioxidant response element
CKMT2-AS1	Creatine kinase, mitochondrial 2 antisense RNA 1
DNMT	DNA methyltransferase
GPX4	Glutathione peroxidase 4
G2/M	G2/M phase of the cell cycle
HDAC	Histone deacetylase
HO-1	Heme oxygenase 1
IL-1β	Interleukin-1 beta
IL-6	Interleukin-6
Keap1	Kelch-like ECH-associated protein 1
MAPK	Mitogen-activated protein kinase
mTOR	Mechanistic target of rapamycin
NADPH	Nicotinamide adenine dinucleotide phosphate
NF-κB	Nuclear factor kappa-light-chain-enhancer of activated B cells
NQO1	NAD(P)H quinone dehydrogenase 1
Nrf2	Nuclear factor erythroid 2–related factor 2
PI3K	Phosphoinositide 3-kinase
ROS	Reactive oxygen species
SFN	Sulforaphane
STAT3	Signal transducer and activator of transcription 3
TRAMP	Transgenic adenocarcinoma of mouse prostate
